# Supramolecular polymer formation by cyclic dinucleotides and intercalators affects dinucleotide enzymatic processing

**DOI:** 10.4155/fso.15.93

**Published:** 2016-01-29

**Authors:** Shizuka Nakayama, Jie Zhou, Yue Zheng, Henryk Szmacinski, Herman O Sintim

**Affiliations:** 1 Department of Chemistry & Biochemistry, University of Maryland, College Park, MD 20742, USA; 2 Department of Chemistry, Purdue University, 560 Oval Drive, West Lafayette, IN 47907, USA; 3 Department of Biochemistry & Molecular Biology, Center for Fluorescence Spectroscopy, University of Maryland School of Medicine, 725 West Lombard St, Baltimore, MD 21201, USA

**Keywords:** c-di-AMP, c-di-GMP, fluorescence lifetime, PDE inhibition, polymer, second messenger, small molecule, supramolecular

## Abstract

**Background:**

Cyclic dinucleotides form supramolecular aggregates with intercalators, and this property could be utilized in nanotechnology and medicine.

**Methods & results:**

Atomic force microscopy and electrophoretic mobility shift assays were used to show that cyclic diguanylic acid (c-di-GMP) forms G-wires in the presence of intercalators. The average fluorescence lifetime of thiazole orange, when bound to c-di-GMP was greater than when bound to DNA G-quadruplexes or dsDNA. The stability of c-di-GMP supramolecular polymers is dependent on both the nature of the cation present and the intercalator. C-di-GMP or cyclic diadenylic acid/intercalator complexes are more resistant to cleavage by YybT, a phosphodiesterase, than the uncomplexed nucleotides.

**Conclusion:**

Cleavage of bacterial cyclic dinucleotides could be slowed down via complexation with small molecules and that this could be utilized for diverse applications in nanotechnology and medicine.

## Abstract (lay abstract)

**Lay abstract:** Bacteria respond to a changing environment and nutrient availability by regulating key metabolic processes. Cyclic dinucleotides are now understood to play pivotal roles in transmitting information about the environment to macromolecular targets, which modulate the bacterial phenotype. In this paper, we demonstrate that these important bacterial second messengers can be aggregated with other small molecules and this process could potentially be used to affect how bacteria sense the environment.

**Figure 1.**
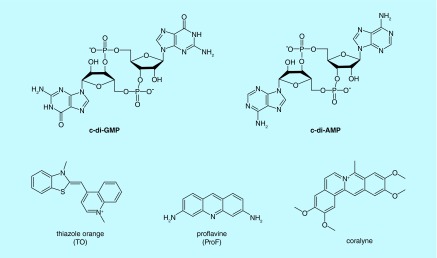
**Structures of cyclic dinucleotides and intercalators.**

**Figure 2.**
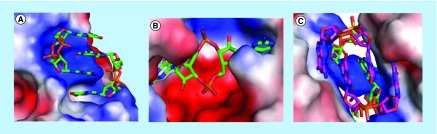
**Cyclic diguanylic acid can bind to proteins as monomers, dimers or tetramers (dimer of a dimer).** **(A)** Crystal structure of cyclic diguanylic acid (c-di-GMP) bound to WspR I-site, DGC protein (protein data bank [PDB] code: 3I5A) [5]; **(B)** Crystal structure of c-di-GMP bound to YahA, PDE protein (PDB code: 4LJ3) [6]; **(C)** Crystal structure of c-di-GMP bound to BldD, transcriptional factor (PDB code: 4OAZ) [7].

**Figure 3.**
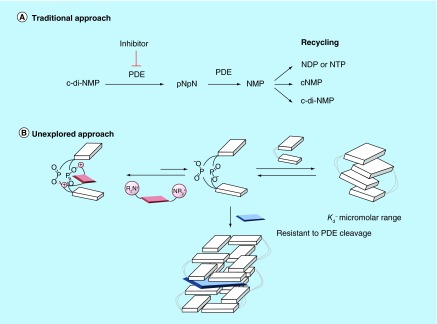
**Different approaches to inhibit dinucleotide hydrolysis.** **(A)** Traditional approach to inhibiting c-di-NMP signaling; **(B)** unexplored approach to inhibiting c-di-NMP signaling via aggregate formation. ‘N’ in c-di-NMP, pNpN, NMP, NDP, NTP or cNMP refers to guanine or adenine base. White block indicates nucleobase. Blue and red shapes represent intercalators. Aggregated forms could be resistant to PDE cleavage. c-di-NMP: Cyclic-di-nucleotide; cNMP: Cyclic nucleotide monophosphate; NDP: Nucleotide diphosphate; NTP: Nucleotide triphosphate; PDE: Phosphodiesterase; pNpN: Linear dinucleotide.

**Figure 4.**
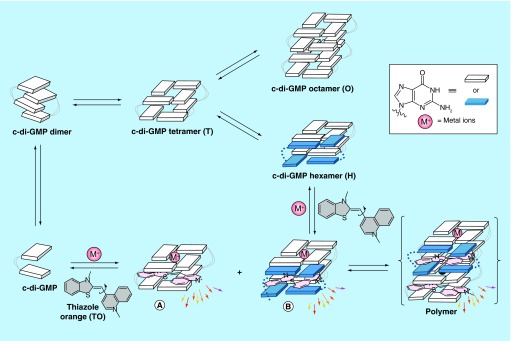
**Proposed cyclic diguanylic acid/intercalator complexes.** **(A)** Tetramer/thiazole orange complex and **(B)** Hexamer/thiazole orange complex. Blue and white indicate guanine, M^+^ represents as metal ion such as K^+^ and colored arrows indicate fluorescence. C-di-GMP can form G-quadruplex structures and intercalators could end-stack or intercalate into these structures. C-di-GMP: Cyclic diguanylic acid.

**Figure 5.**
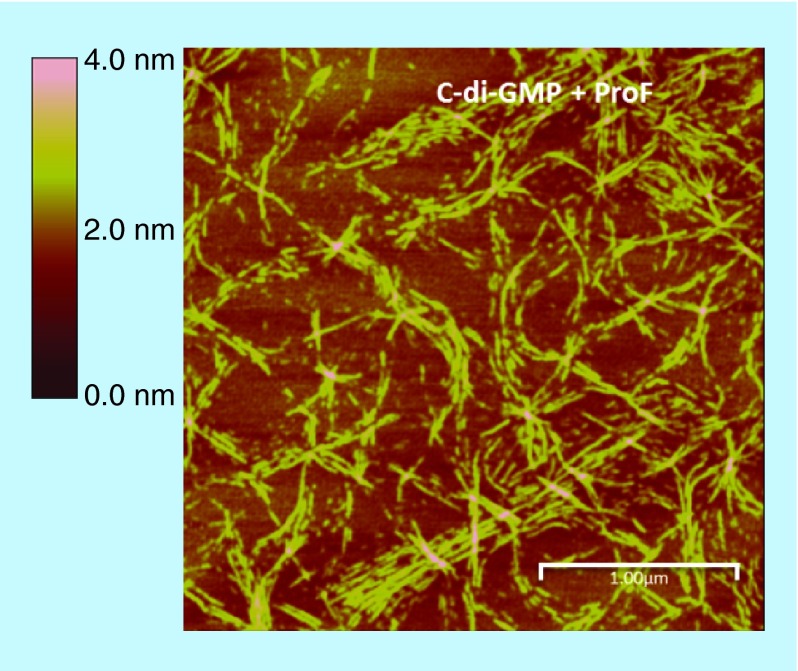
**Complex formation between cyclic diguanylic acid and 3,6-diaminoacridine hydrochloride captured by atomic force microscopy image.** Condition: [c-di-GMP] = 100 μM, [ProF] = 100 μM, [K^+^] = 250 mM in 50 mM Tris-HCl (pH = 7.5) was left to stand overnight (12 h) and then diluted 20-times to give final concentrations of c-di-GMP (5 μM), ProF (5 μM) and K^+^ (12.5 mM), which was deposited on the mica plate for AFM imaging. AFM: Atomic force microscopy; c-di-GMP: Cyclic diguanylic acid; ProF: 3,6-Diaminoacridine hydrochloride.

**Figure 6.**
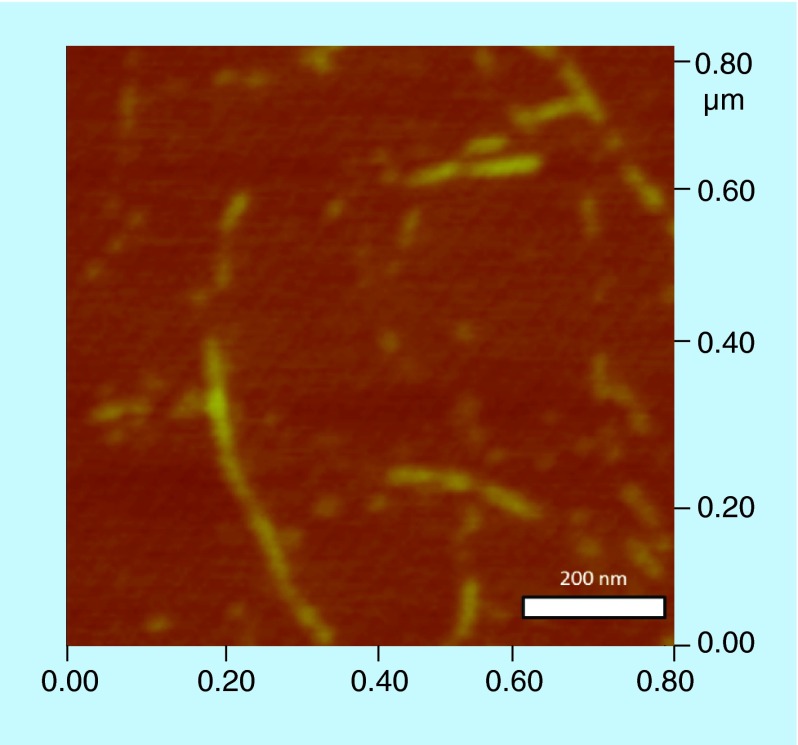
**Cyclic diguanylic acid/3,6-diaminoacridine hydrochloride polymer could be as long as 0.4 microns.**

**Figure 7.**
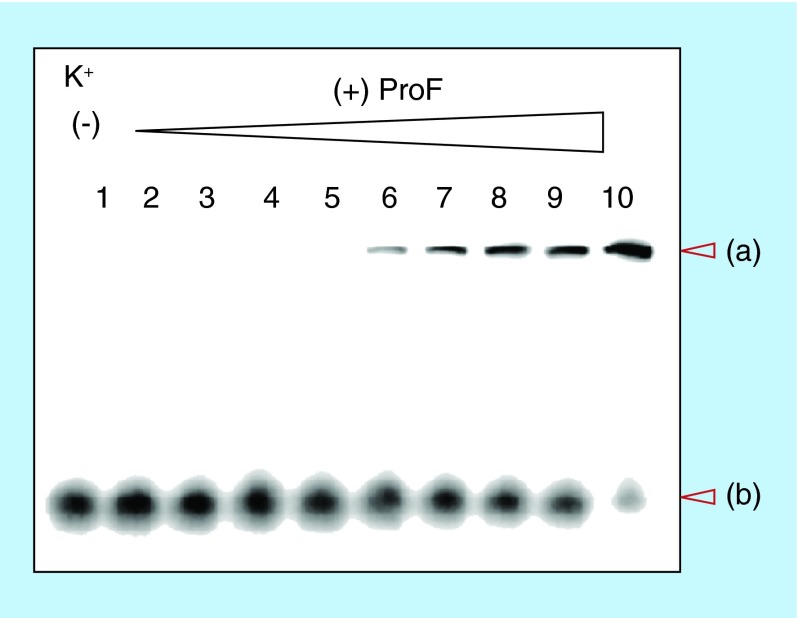
**Electrophoretic mobility shift assays using native gel.** 100 μM unlabeled cyclic diguanylic acid (c-di-GMP) and 0.8 nM ^32^P-c-di-GMP were incubated with various concentrations of ProF in the presence of 250 mM K^+^. Final concentration of ProF: lane 1 = 0 μM; lane 2 = 1 μM; lane 3 = 5 μM; lane 4 = 10 μM; lane 5 = 15 μM; lane 6 = 20 μM; lane 7 = 30 μM; lane 8 = 40 μM; lane 9 = 50 μM and lane 10 = 100 μM. **(a)** = high molecular weight aggregate and **(b)** = monomeric c-di-GMP. ProF: 3,6-Diaminoacridine hydrochloride.

**Figure 8.**
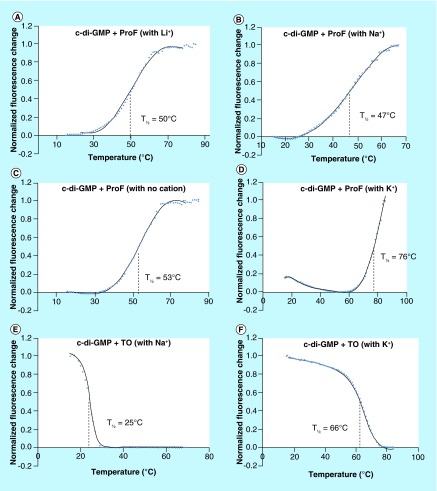
**Fluorescence melting of the complex formed between cyclic diguanylic acid and thiazole orange or 3,6-diaminoacridine hydrochloride in the presence of different cations.** Condition: [c-di-GMP] = 100 μM, [ProF or TO] = 10 μM, [Na^+^, K^+^ or Li^+^] = 250 mM in 50 mM Tris-HCl (pH = 7.5). The pH of Tris is known to slightly fluctuate with temperature. As a control, melting experiment was also performed in MOPS buffer and similar melting trend was also observed, see Supplementary Figure 6. The melting of ProF itself as control was included in Supplementary Information (see Supplementary Figure 7). **(A)** Melting of c-di-GMP/ProF polymer in the presence of Li^+^; **(B)** melting of c-di-GMP/ProF polymer in the presence of Na^+^; **(C)** melting of c-di-GMP/ProF polymer without added cation; **(D)** melting of c-di-GMP/ProF polymer in the presence of K^+^; **(E)** melting of c-di-GMP/TO polymer in the presence of Na^+^; **(F)** melting of c-di-GMP/TO polymer in the presence of K^+^. c-di-GMP: Cyclic diguanylic acid; ProF: 3,6-Diaminoacridine hydrochloride; TO: Thiazole orange.

**Figure 9.**
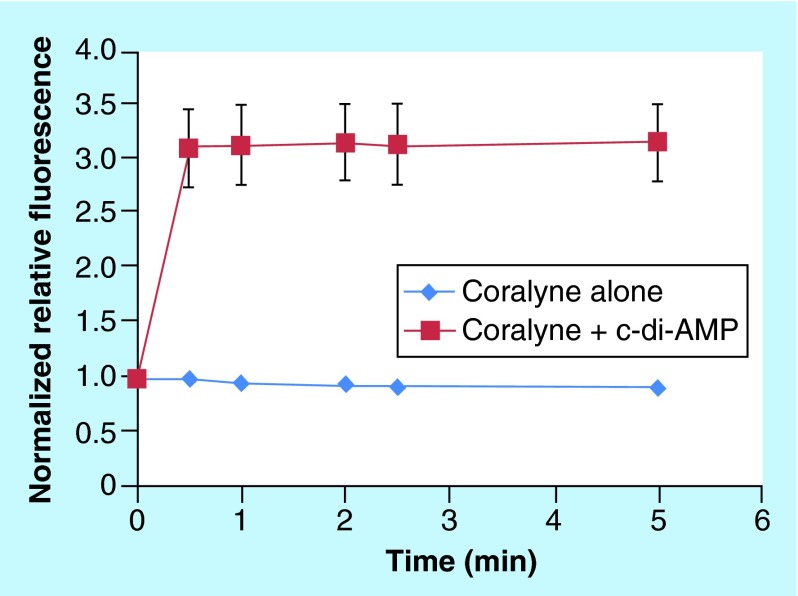
**Kinetics of cyclic diadenylic acid/coralyne complex formation.** Condition: C-di-AMP (10 μM), KI (3 mM) and buffer (50 mM Tris-phosphate [pH 7.5]) were heated up at 95°C for 5 min. Coralyne (10 μM) was then added and mixed well. The sample was subject to fluorescence monitoring at 25°C over 5 min. Excitation: 420 nm and emission: 475 nm. C-di-AMP: Cyclic diadenylic acid.

**Figure 10.**
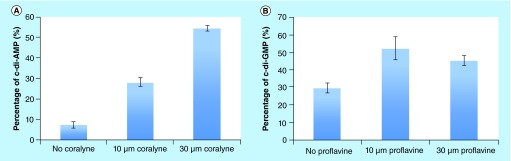
**Intercalator-mediated dinucleotide aggregation inhibits c-di-NMP cleavage.** **(A)** YybT cleavage of ^32^P-c-di-AMP in the presence and absence of coralyne. **(B)** YybT cleavage of ^32^P-c-di-GMP in the presence and absence of ProF. Condition: [YybT] = 1.5 μM, [c-di-GMP or c-di-AMP] = 10 μM (^32^P labeled + unlabeled), [coralyne or ProF] = 10 or 30 μM, [KCl] = 10 mM in **(B)**. **(A)** was done at 37°C for 20 min and **(B)** was done at 37°C for 30 min. c-di-AMP: Cyclic diadenylic acid; c-di-GMP: Cyclic diguanylic acid; ProF: 3,6-Diaminoacridine hydrochloride.

Cyclic diguanylic acid (c-di-GMP) and cyclic diadenylic acid (c-di-AMP; see Figure 1) have emerged as important second messengers in bacteria and control diverse processes, such as biofilm formation, antibiotic resistance and cell wall synthesis [1]. Due to the many processes that these second messengers regulate, it is highly likely that the interruption of c-di-GMP or c-di-AMP signaling could adversely affect bacterial fitness. Consequently, strategies to interrupt cyclic dinucleotide signaling are desired. So far most approaches to interrupt cyclic dinucleotides have focused on the inhibition of metabolism and regulatory proteins and RNA [1]. In this paper, we characterize supramolecular formation by c-di-AMP and c-di-GMP and explore the possibility of inhibiting dinucleotide hydrolysis by phosphodiesterases (PDEs) via dinucleotide aggregate formation (and not via the classical enzyme inhibition), *vide infra*.

Cyclic dinucleotides exhibit interesting polymorphism in solution [2–4] or when bound to proteins (see Figure 2) [5–8]. In the last few years, we have provided many examples of c-di-GMP or c-di-AMP supramolecular aggregate formation in the presence of aromatic intercalators, such as thiazole orange (TO) [3] or coralyne [9] (see Figure 1), and we now demonstrate herein that beyond sensing applications, the propensity of c-di-GMP and c-di-AMP to readily form supramolecular aggregates could be utilized in nanobiotechnology or provocatively could affect microbiota. Indeed supramolecular aggregation or gelation by nucleotides is not a new phenomenon and nucleic acid-mediated gelation has found use in widespread applications, including analyte sensing and the fabrication of smart materials [10,11]. Guanine gels, formed from guanine nucleotides have been known since the early 60s, when Davis and coworkers showed that GMP could form gels in water [12]. Subsequent works revealed that the guanine gels were made of sheets of G-tetrad [13–16] or pseudo-four-stranded helix, maintained by guanine–guanine H-bonding [13,17]. In the ensuing years, others have reported sophisticated nanostructures made of guanosine monomers [18]. Some of these guanosine-based supramolecular aggregates have been used for sensing purposes [19] or as ionophores [20]. In prior works related to G-gel formation in water, high concentrations of guanine nucleotides (millimolar to molar) have been used to form the supramolecular aggregates [21]. In 1990, Wang made an important observation that the bacterial second messenger, c-di-GMP, affected the UV absorption profiles of a few planar intercalators [22]. It took two decades before some experimental data were provided by others to support a plausible G-quadruplex formation by c-di-GMP at low micromolar concentrations in the presence of intercalators, such as ProF (3,6-Diaminoacridine hydrochloride) and TO (see structures in Figure 1) [3,23–25]. Circular dichroism spectra of c-di-GMP in the presence and absence of intercalators provided circumstantial evidence that c-di-GMP could form G-quadruplexes at low micromolar concentrations in water, in the presence of intercalators [3,23,25]. Attempts to provide a more definitive proof for c-di-GMP G-quadruplex formation at physiologically relevant concentrations (˜10 μM) [26] using nuclear magnetic resonance (NMR) failed; although the addition of ProF to c-di-GMP caused the disappearance of monomer and dimer c-di-GMP NMR peaks, no new peaks around the imino proton region (˜11 ppm) appeared and so G-quadruplex formation could not be confirmed [4]. The lack of signals around the imino proton region in the NMR spectrum of c-di-GMP/ProF does not necessarily rule out G-quadruplex formation. It is known that G-quadruplex DNA can aggregate to form large complexes that exhibit NMR line broadening (due to relaxation effects) [27]. Second, such large G-quadruplexes could precipitate or ‘crash out’ of the NMR solution.

It remains to be determined if c-di-GMP can form large supramolecular aggregates with intercalators and if such aggregation could affect the biological processing of c-di-GMP. Recently, we revealed that another important bacterial signaling molecule, c-di-AMP, also form supramolecular structures in the presence of coralyne [9]. It, therefore, appears that perhaps a simple yet unexplored strategy to modulate the signaling of cyclic dinucleotide second messengers is to affect the signal itself (via aggregation), rather than inhibit the processing enzymes (synthases and PDE) or receptor proteins and RNA that bind to the nucleotide second messengers (the classic strategy used to intercept signaling), see Figure 3. Beyond a plausible biological implication for cyclic dinucleotide aggregation, if simple nucleotides, such as c-di-GMP and c-di-AMP could form well-defined supramolecular fluorescent aggregates at low micromolar concentrations in water, then this phenomenon could be tapped into for biotechnological and medical applications [28–32]. In a beautiful demonstration of DNA programmability, Armitage and coworkers reported interesting DNA G-wires made up of alternating nucleobase/fluorophore units (fluorescent DNA nanotags) [33]. Conceivably, c-di-GMP or c-di-AMP and fluorophore could form fluorescent nucleotide nanotags that might have interesting optical or electrical properties. Herein, we use a panel of biophysical and biochemical methods, including fluorescence lifetime, atomic force microscopy (AFM), gel shift assays and PDE cleavage assay to show that not only does intercalators facilitate the aggregation of cyclic dinucleotides but also the hydrolysis of c-di-GMP and c-di-AMP can be inhibited with small molecules via dinucleotide aggregation (which is different from the classic enzyme inhibition).

## Results & discussion

C-di-GMP is known to form a tetramer (T, Figure 4) from four monomers. The internucleobase distance between the G-tetrads in the tetramer form is approximately 6.8 Å. Because this distance is larger than 3–4 Å, distances that allow for effective π–π stabilization of the G-tetrad planes (typical interplanar distances in DNA or RNA G-quadruplexes ranges from 3.4 to 4.7 Å) [34], π–π interaction is not optimal in the tetramer form. Upon interdigitization of the tetramer by four additional monomers, an octamer (O, Figure 4) is formed. If a tetramer is present as an intermediate, then it is plausible that an intercalator of a right size could move into the cavity of the tetramer, instead of the c-di-GMP nucleobase. The intercalator would then stabilize the tetramer via π–π stacking to both G-tetrad planes of the tetramer to form complex A (Figure 4) [35,36]. Alternatively, if the tetramer is converted into an octamer via the sequential additions of c-di-GMP monomers, then a hexamer (H, Figure 4) should exist in the pathway. At the hexamer stage, an intercalator could occupy the remaining space between the G-tetrad planes, instead of c-di-GMP, forming hexamer/intercalator complex (B, Figure 4). Complex A or B can then polymerize into G-wires using c-di-GMP or intercalator monomers via the pathway proposed by Spada and Mariani [15].

### Is there any evidence for c-di-GMP G-wire formation?

In our polymerization model (Figure 4), we posited that a G-wire could form from c-di-GMP and intercalators. To provide a direct evidence for G-wire formation we used AFM to image the polymer that is formed when c-di-GMP is mixed with ProF, following Henderson's precedent [37]. AFM studies revealed that in the presence of potassium, ProF, c-di-GMP or GTP did not form any fibers on mica. Interestingly, AFM image revealed fibers for c-di-GMP/ProF complex (but not GTP/ProF complex), see Figure 5, Supplementary Figure 1 & Supplementary Figure 2. The c-di-GMP/ProF fiber could be as long as 0.4 microns (see Figure 6). When ProF was used as an intercalator, fiber formation by c-di-GMP could also occur in the presence of Li^+^ or Na^+^ (see Supplementary Figure 1). This contrasted with when TO was used, where fiber formation was mainly seen when K^+^ or Na^+^ (only a few fibers), but not Li^+^, was present (see Supplementary Figure 3). It appears that the addition of intercalators and cations to c-di-GMP leads to bundles (see AFM Figure 5). Others have also observed bundles of G-tetrad rods [38]. It has been hypothesized that these bundles arise from the interactions of cations between the phosphate moieties of adjacent rods [38].

We used electrophoretic mobility shift assays (EMSA, see Figure 7, Supplementary Figure 4 & Supplementary Figure 5) to confirm the AFM data that c-di-GMP can indeed form submicron polymers. EMSA or gel retardation assays are typically used to investigate size, charge or shape (to a lesser extent) changes after molecular interactions. Lower molecular weight complexes or monomers can move through a polyacrylamide gel faster than higher molecular weight aggregates that result from molecular associations. For a gel shift assay to work, the noncovalent aggregate should be stable enough to withstand the EMSA process. We synthesized radiolabeled c-di-GMP (^32^P-c-di-GMP, referred to as ‘hot’ c-di-GMP hereafter) from ^32^P-GTP, using WspR D70E as the synthase. The hot c-di-GMP (0.8 nM) was mixed with cold c-di-GMP (100 μM) and incubated with ProF or TO in the absence or presence of various monovalent cations (Li^+^, Na^+^ or K^+^). Addition of ProF to c-di-GMP resulted in the appearance of a very high molecular weight polymer that barely moved out the wells of the gel after 30 min of gel electrophoresis (see Figure 7). The EMSA data confirmed the AFM data; c-di-GMP/ProF aggregate could form in the presence of Li^+^ or even when no cation was present (Figure 7). Also, the EMSA data confirmed that for TO, only Na^+^ or K^+^ could cause higher order aggregation (see Supplementary Figure 4). Intercalator-mediated polymer formation in the presence of K^+^ or Na^+^ but not Li^+^ provides a circumstantial but strong support for the involvement of G-quadruplexes in these polymers. This also augments earlier indirect circular dichroism (CD) evidence for G-quadruplex involvement in c-di-GMP–intercalator complexes [25].

We were somehow surprised that noncovalent nucleotide supramolecular aggregates (such as c-di-GMP/ProF or TO) could withstand gel electrophoresis. We, therefore, proceeded to gain insights into the stability of these polymers using melting experiment to obtain T_1/2_ (temperature at which 50% of the aggregate becomes dissociated, see Figure 8). The melting experiment reaffirmed our earlier observations that ProF, but not TO, can form strong complexes with c-di-GMP in the presence of Li^+^ or even when no cation is present and that complexes formed when K^+^ is present are the strongest. Potassium also promotes the formation of stable RNA and DNA G-quadruplex complexes [23], but the ability of ProF to facilitate c-di-GMP to form G-wire-like fibers in the presence of Li^+^ or even when no cation is presented is somehow unique to c-di-GMP. EMSA analysis of c-di-AMP in the presence of coralyne failed to reveal higher order structures. We conclude that c-di-GMP/intercalator complexes are more stable than c-di-AMP/intercalator complexes.

When c-di-GMP binds to ProF, the fluorescence of ProF is quenched. On the other hand c-di-GMP enhances the fluorescence of TO. We were interested to know if the binding environment of TO in the c-di-GMP aggregate was similar to DNA G-quadruplex or duplex DNA [39,40]. Therefore, we investigated the fluorescence lifetime of TO bound to c-di-GMP, c-Myc and hTel G-quadruplexes and duplex DNA (see Table 1). We have previously shown that the fluorescence intensity of TO when bound to c-di-GMP is highest when the cation present is sodium [3]. Therefore, we used Na^+^ for all of the lifetime studies. Theoretically, four c-di-GMP molecules are needed to form a G-quadruplex and so we used 100 μM c-di-GMP but only 25 μM DNA for the lifetime studies (4:1). Since fluorescent lifetime is invariant of concentration, it was nonetheless inconsequential to whatever concentrations that we used. Interestingly, the fluorescence lifetime of TO when bound to c-di-GMP was the highest τ_avg_ = 6.06 and 4.72 ns at high (1 M) and medium (100 mM) Na^+^ concentrations, respectively, compared with 2.88 and 3.64 ns (hTel), 2.19 and 2.18 ns (c-Myc), 1.27 and 1.26 ns (double stranded DNA; dsDNA). Algar *et al*. reported that the fluorescence lifetime for TO bound to a 19bp dsDNA, under a different condition to ours, is 2.6 ns [41]. Reported values are close to ours when using single exponential fit of 2.33 ns; however, we found that intensity decays of TO/dsDNA are heterogeneous as shown in large improvement in χ^2^_R_ of fitting from one- and two-exponential model. Based on the fluorescence lifetime data, and simultaneously measured intensity values (Table 1), we conclude that the binding environment of TO is significantly different in c-di-GMP aggregate, G-quadruplexes and dsDNA.

### Is the formation of c-di-GMP or c-di-AMP polymers a fast process?

It is known that nucleic acid-based supramolecular polymers are formed in an extremely slow pace (10–24 h) [42]. However, whereas the aggregation of c-di-GMP with ProF is slow (data not shown), that of c-di-AMP with coralyne is fast. When 10 μM coralyne was added to 10 μM c-di-AMP, complex formation was observed in <1 min, see Figure 9.

### Does intercalator-mediated aggregation of c-di-GMP or c-di-AMP affect enzymatic processing?

Nucleotides are also used for the synthesis of other biopolymers and as second messengers to control other processes. The accumulation of only one type of nucleotide could, therefore, adversely affect the cell if there are no other means to recycle the nucleotide. Therefore, PDEs do not only ‘quench’ the nucleotide signals but also aid in the recycling of second messenger nucleotides. Cyclic dinucleotide signaling systems have feedback mechanisms that control the synthesis of a particular cyclic dinucleotide [5,6]. Conceivably, the sequestration of c-di-GMP or c-di-AMP with aromatic intercalators would affect the feedback mechanism and could lead to the depletion of ATP or GTP pools. Several drugs contain aromatic heterocyclic moieties that could potentially interact with c-di-GMP or c-di-AMP. For example, acridine-based drugs have been used as anticancer [43] or antibacterial [44] agents, and for these applications, it has been assumed that biological effect or clinical efficacy is derived (at least in part) from DNA intercalation. The demonstration that these heterocyclic aromatic molecules can interact with bacterial signaling molecules hints at possible interference of microbial physiology via cyclic dinucleotide aggregation. To provide some evidence that this could be possible, at least in principle, we investigated the cleavage of c-di-AMP and c-di-GMP by a cyclic dinucleotide-specific PDE YybT, which is able to cleave c-di-AMP into its linear form, 5’-pApA [45]. YybT (also named GdpP) is also able to cleave c-di-GMP, albeit slower than cleavage of c-di-AMP [45]. As shown in Figure 10A, if there was no coralyne present, >90% c-di-AMP was cleaved by YybT in 30 min. When 10 μM coralyne was added, cleavage was slightly slowed down. However, when 30 μM coralyne was added into the cleavage assay, only 40% c-di-AMP was cleaved by YybT (compared with 90% cleavage when no coralyne was added). Increasing the concentration of coralyne further to 100 μM led to 90% of cleavage inhibition (data not shown). Although it would be difficult to achieve such a high level of intracellular concentration of coralyne (100 μM), this nonetheless validates the hypothesis that intercalators can be used to shut off cyclic dinucleotide processing. To eliminate the possibility that the coralyne inhibition of YybT cleavage of c-di-AMP was not due to direct inhibition of the enzyme, we added 100 μM coralyne to a mixture containing YybT and c-di-GMP (see Supplementary Figure 8). Coralyne does not aggregate c-di-GMP and unlike the case of c-di-AMP, coralyne did not inhibit the cleavage of c-di-GMP by YybT, confirming that the coralyne did not directly inhibit YybT but rather aggregated c-di-AMP and ‘protected’ it from PDE cleavage. Similarly, c-di-GMP cleavage by YybT was inhibited when the c-di-GMP was first incubated with ProF (Figure 10B). ProF did not inhibit the cleavage of c-di-AMP by YybT, implying that with this case also the inhibition was not a direct enzymatic inhibition but rather via the ‘protection’ of c-di-GMP from PDE cleavage.

### Limitations of the use of intercalators to interrupt c-di-NMP signaling *in vivo*

We have demonstrated that at least in principle, it is possible to use small molecules to interrupt c-di-NMP signaling at physiologically relevant c-di-AMP or c-di-GMP concentrations (10 μM) [26]. C-di-GMP can be aggregated not only with acridines but also other small molecules, such as diminazene, which are not as mutagenic as acridines [46]. However, because the association kinetics of c-di-GMP (but not c-di-AMP) and intercalators is slow, it appears that this approach might be better suited for interrupting c-di-AMP signaling and not c-di-GMP. Plausibly, a coralyne-type molecule that also takes advantage of other noncovalent interactions with c-di-AMP, such as utilizing establishing a salt bridge between the intercalator and the anionic phosphates, could have a higher inhibitory profile and could find practical application. It is important that any molecule that is designed to interrupt bacterial-derived cyclic dinucleotides does not interfere with the human cyclic dinucleotides, such as 2′3′-cGAMP [47]. We are currently working on such molecules and will report on them in due course. Perhaps the most likely application of the supramolecular aggregate formation or potential gelation by bacterial-derived cyclic dinucleotides is in bionanotechnology.

## Materials & methods

### General experimental methods

ProF (3,6-Diaminoacridine hydrochloride) was purchased from Sigma-Aldrich (MO, USA). TO was purchased from Sigma-Aldrich. C-di-GMP and c-di-AMP were synthesized following literature and obtained as triethylammonium acetate salt [48]. ^32^P-GTP or ^32^P-ATP was purchased from Perkin Elmer (MA, USA). Images were obtained by STORM scanner and quantified by ImageQuant software (Molecular Dynamics). Fluorescence was measured by Cary Eclipse Fluorescence Spectrophotometer. AFM image was taken by Veeco Multimode AFM with nanoscope III controller with tapping mode. The visualization probe was Silicon AFM Probes TAP 300 from Ted Pella, Inc. Fluorescence lifetime was measured using time-domain system integrated with fluorescence lifetime imaging microscope (FLIM) system Alba V (ISS, IL, USA). The system is equipped with SPC-830 TCSPC module and pulsed laser system (Becker and Hickl GmbH). Laser BHL-473 nm and observation through band pass filter 514/50 nm was used for TO. Data analysis was performed using Vista Vision software v. 218 from ISS.

### Synthesis of ^32^P-c-di-GMP & ^32^P-c-di-AMP

^32^P-c-di-GMP and ^32^P-c-di-AMP used in the experiments were synthesized from 33 nM ^32^P-NTP (NTP = GTP or ATP), catalyzed by 40 μM WspR D70E (*Pseudomonas aeruginosa*. The D70E substitution mimics the phosphorylated, presumably active, conformation of WspR. WspR is, therefore, constitutively active) or DisA (*Bacillus subtilis*) at 37°C overnight. The buffer for WspR D70E reaction contains 10 mM Tris-HCl, pH 8.0, 100 mM NaCl and 5 mM MgCl_2_ and buffer for DisA reaction contains 40 mM Tris-HCl, pH 7.5, 100 mM NaCl and 10 mM MgCl_2_. After incubation, reaction mixture was heated up to 95°C and kept at 95°C for 5 min and cooled down to room temperature in 10 min, followed by filtration through 10 KD filter. The conversion was analyzed by a cellulose TLC (EMD Millipore, MA, USA) with running solvent 1:1.5 (v/v) saturated (NH_4_)_2_SO_4_ : 1.5 M KH_2_PO_4_.

### Preparation of native gel

The following components and quantities were used to prepare the native gel: 5 × Tris/Borate/ethylenediaminetetraacetic acid (TBE, 5 ml), 40% acrylamide/bisacrylamide (19:1; 5 ml), 80% glycerol (625 μl), 10% ammonium persulfate (APS; 200 μl), water (9.1 ml) and N,N,N,N’-tetramethylenediamine (TEMED; 20 μl). For running buffer, 0.5 × TBE containing 50 mM of cation (for each experiment) was used. The running temperature for gel was room temperature under 140 V for 40 min. The gel was dried by gel dryer model 583 (Bio-Rad, CA, USA) for 2 h under vacuum.

### Sample preparation for native gel using ^32^P labeled c-di-GMP

100 μM unlabeled c-di-GMP, 0.8 nM ^32^P-c-di-GMP, 50 mM Tris-HCl (pH 7.5) were mixed with different cations and intercalators with concentrations described in figure legend for experiments. Unlabeled c-di-GMP, ^32^P-c-di-GMP and cation were first mixed in buffer and heated up at 95°C for 5 min and cooled down to room temperature for 15 min. Then intercalator was added and incubated at room temperature for approximately 12 h. Before running a gel, 2 μl loading dye was added into 5 μl sample mixture. Loading dye for native gel was prepared as 6 × stock solution (50% glycerol, 33 mM Tris-HCl (pH 8.0), 0.15% (v/w) bromophenol blue).

### Fluorescence melting

100 μM c-di-GMP in 50 mM Tris-HCl (pH 7.5) or MOPS buffer, containing different concentrations of cation as described in figure legend for each experiment, was heated up to 95°C and kept there for 5 min. The mixture was allowed to cool down to room temperature in 15 min. Then 10 μM intercalator was added and incubated for approximately 12 h at 4°C. Fluorescence melting experiment was then conducted on a Cary Eclipse Fluorescence Spectrophotometer.

### Sample preparation for AFM measurement

Mica was used to obtain AFM image. It was cleaved immediately before use and pretreated with 10 mM MgCl_2_ for 3 min and washed with distilled deionized filtered water (4 ml). After drying, sample (20 μl) was deposited onto Mica. Sample preparation was done with 100 μM c-di-GMP, 100 μM ProF, 50 mM Tris-HCl (pH 7.5) containing 250 mM KCl, incubated at 24°C for 12 h. Before deposition, the sample mixture was diluted with 10 mM Tris-HCl (pH 7.5) containing 10 mM MgCl_2_. After 30 min incubation, the mica surface was washed with 4 ml water and dried by air.

### Protein purification & PDE cleavage assay

WspR D70E (*P. aeruginosa*), DisA (*B. subtilis*) and YybT (*B. subtilis*) were purified as described previously [49]. 1.5 μM YybT reacted with 10 μM c-di-GMP (^32^P labeled + unlabeled) at 37°C for 30 min. 1.5 μM YybT reacted with 10 μM c-di-AMP (^32^P labeled + unlabeled) at 37°C for 20 min. YybT reaction buffer contains 100 mM Tris-HCl, pH 8.3, 20 mM KCl, 1 mM DTT and 0.5 mM MnCl_2_. Intercalators and cations were added where indicated. Reactions were quenched by heating up at 95°C for 5 min. 0.3 μl reaction mixture was applied on a cellulose TLC (EMD Millipore). TLC was developed in a buffer containing 1.5:1 (v/v) 1.5 M KH_2_PO_4_ and saturated (NH_4_)_2_SO_4_.

### Sample preparation for fluorescence lifetime measurement

100 μM c-di-GMP or 25 μM DNAs, 100 mM or 1 M Na^+^ and 50 mM Tris-HCl (pH 7.5) were first heated up at 95°C for 5 min without adding intercalator. After cooling down to room temperature, 3 μM TO was added into the mixture and incubated at 4°C for approximately 12 h.

### Kinetics assay

10 μM c-di-AMP, 3 mM KI and 50 mM Tris-phosphate (pH 7.5) were heated up at 95°C for 5 min. 10 μM coralyne was then added and mixed well. The sample was subject to fluorescence monitoring at 25°C over 5 min with excitation at 420 nm and emission at 475 nm.

## Conclusion

Several studies have confirmed that aromatic intercalators can associate with c-di-GMP or c-di-AMP, but the nature of the complex or complexes formed between c-di-GMP or c-di-AMP with intercalators remains to be characterized. Intercalators are known to bind to DNA or RNA and can prevent the enzymatic processing of nucleic acids [50]. Although analogously intercalators could also affect the enzymatic processing of cyclic dinucleotides or the perception of these second messengers by effector molecules, this had not been demonstrated. In this manuscript, we used AFM and EMSA to demonstrate that aromatic intercalators facilitate fiber formation by c-di-GMP. We also demonstrate that supramolecular aggregate formation by c-di-GMP or c-di-AMP in the presence of intercalators can indeed inhibit the hydrolysis of these important signaling molecules by enzymes. C-di-GMP has been shown to affect bacterial biofilm formation whereas c-di-AMP affects several processes, including bacterial cell wall formation, and it is plausible that the aggregation of these molecules could also inhibit the aforementioned processes [1]. Several challenges, such as toxicity of intercalators, need to be addressed before the proposed intercalator-mediated aggregation of cyclic dinucleotides could be used to control bacterial infection in humans. However, there are other scenarios, such as the sterilization of hospital instruments or surfaces, whereby the genotoxicity of these intercalators would not be an issue and hence the proposed approach could have traction. Additionally, this paper also suggests that perhaps an unintended or underappreciated consequence of taking acridine-based medications or drugs that contain planar motifs is that human microbiota could be affected via the modulation of cyclic dinucleotide signaling. Interestingly, this work also demonstrates that cyclic dinucleotides could compete with DNA and RNA for intercalator binding and that medicinal chemists should be aware of this fact when designing nucleic acid-based drugs as antibacterial agents.

## Future perspective

We have provided data to support G-wire formation by c-di-GMP, facilitated by small molecules. Additionally, we have shown that the enzymatic processing of cyclic dinucleotides can be affected by intercalators and this provides a tantalizing or provocative strategy to affect bacterial physiology via second messenger ‘protection’ and not via the classical enzyme inhibition strategy. For this new approach to become practical, the following conditions must be met: the cyclic dinucleotide aggregators should ideally not bind to nucleic acids (DNA or RNA), as this reduces the free molecule that will be available to interact with the cyclic dinucleotide. Also, DNA interactive ligands are typically mutagenic; the association kinetics between the small molecule and c-di-GMP or c-di-AMP should be fast; and the ratio of c-di-GMP or c-di-AMP to aggregator and stability of the complex should be high so that low digit or submicromolar concentrations can be used to achieve meaningful processing inhibition.

**Table 1:** **Intensity decay analysis of thiazole orange.**

**Thiazole orange**	**τ_i_ (ns)**	**f_i_**	**τ_avg_ (ns)^†^**	**χ^2^_R_**	**Relative intensity^‡^**	**Relativeτ_avg_**
Water	2.63, 0.98, 4.57	1, 0.943, 0.057	2.63, 1.19	1.9, 1.24	0.85	1.59
1 M Na^+^	2.47, <0.70>, 3.86	1, 0.984, 0.016	2.47, 0.75	2.9, 1.43	1.0	1.0
1 M Na^+^ and 100 μM c-di-GMP	5.85, 4.21, 7.07	1, 0.354, 0.646	5.85, 6.06	4.2, 1.20	2445	8.08
1 M Na^+^ and 25 μM hTel	3.94, 1.72, 5.04	1, 0.649, 0.351	3.94, 2.88	15.0, 1.32	530	3.84
1 M Na^+^ and 25 μM c-Myc	3.54, 1.46, 5.07	1, 0.798, 0.202	3.54, 2.19	9.9, 1.47	315	2.92
1 M Na^+^ and 25 μM dsDNA	2.33, 1.02, 3.06	1, 0.877, 0.123	2.33, 1.27	12.3, 1.53	370	1.69
100 mM Na^+^	2.35, <0.70>, 3.96	1, 0.986, 0.014	2.35, 0.75	3.1, 1.91	0.8	1.0
100 mM Na^+^ and 100 μM c-di-GMP	5.24, 2.56, 6.46	1, 0.447, 0.553	5.24, 4.72	11.3, 1.06	1380	6.29
100 mM Na^+^ and 25 μM hTel	4.60, 1.97, 5.85	1, 0.569, 0.431	4.60, 3.64	15.2, 1.29	740	4.85
100 mM Na^+^ and 25 μM c-Myc	3.53, 1.45, 5.03	1, 0.797, 0.203	3.53, 2.18	19.2, 1.75	340	2.91
100 mM Na^+^ and 25 μM dsDNA	2.32, 1.01, 3.06	1, 0.879, 0.121	2.32, 1.26	13.5, 1.78	590	1.68

^†^Average lifetime τ_avg_ = Σ f_i_ τ_i_, where the τ_i_ and f_i_ are decay times and fractional intensities, respectively.

^‡^Intensity measured at excitation 473 nm with band pass filter 514/50 nm, normalized to intensity for sample TO in 1M Na^+^. Condition: [c-di-GMP] = 100 μM, [c-di-GMP] = 25 μM, [TO] = 3 μM, [Na^+^] = 100 mM or 1 M. hTel sequence (5′–3′): AGGGTTAGGGTTAGGGTTAGGG; c-Myc sequence (5′–3′): TGGGGAGGGTGGGGAGGGTGGGGA. dsDNA sequence (5′–3′): CGAATTTCAAAAGAAATTCG.

**Executive summary**

Bacterial cyclic dinucleotides, cyclic diguanylic acid (c-di-GMP) and cyclic diadenylic acid (c-di-AMP), associate with small molecules to form higher order aggregates.
The fluorescence lifetime of thiazole orange when bound to c-di-GMP is higher than when bound to DNA G-quadruplex or duplex DNA.
Such small molecules-induced c-di-GMP or c-di-AMP supramolecular formation affects the enzymatic processing of these second messengers.
For practical *in vivo* application in humans, any small molecule that aggregates c-di-AMP or c-di-GMP should not affect human endogenous cGAMP.
