# Supplementary material for: Supramolecular polymer formation by cyclic dinucleotides and intercalators affects dinucleotide enzymatic processing
Source: Future Sci OA. 2016 Jan 29;2(1):FSO93. doi: 10.4155/fso.15.93 (PMC5137846; doi:10.4155/fso.15.93)
Supplement: Supplementary file 1 [file fso-02-93-s1.doc]

**Supporting Information**

**Sample preparation for AFM measurement in Figure S1 to S3.**

Mica was used to obtain AFM image. It was cleaved immediately before use and pretreated with 10 mM MgCl_2_ for 3 min and washed with distilled deionized filtered water (4 mL). After drying, sample (20 μL) was deposited onto Mica. Sample preparation was done with 100 μM c-di-GMP, 100 μM proflavine (ProF) or thiazole orange (TO), 50 mM Tris-HCl (pH 7.5) containing 250 mM LiCl, NaCl, KCl or no added cation incubated at 24 ⁰C for 12 hr. Before deposition, the sample mixture was diluted with 10 mM Tris-HCl (pH 7.5) containing 10 mM MgCl_2_. After 30 min incubation, the mica surface was washed with 4 mL water and dried by air.


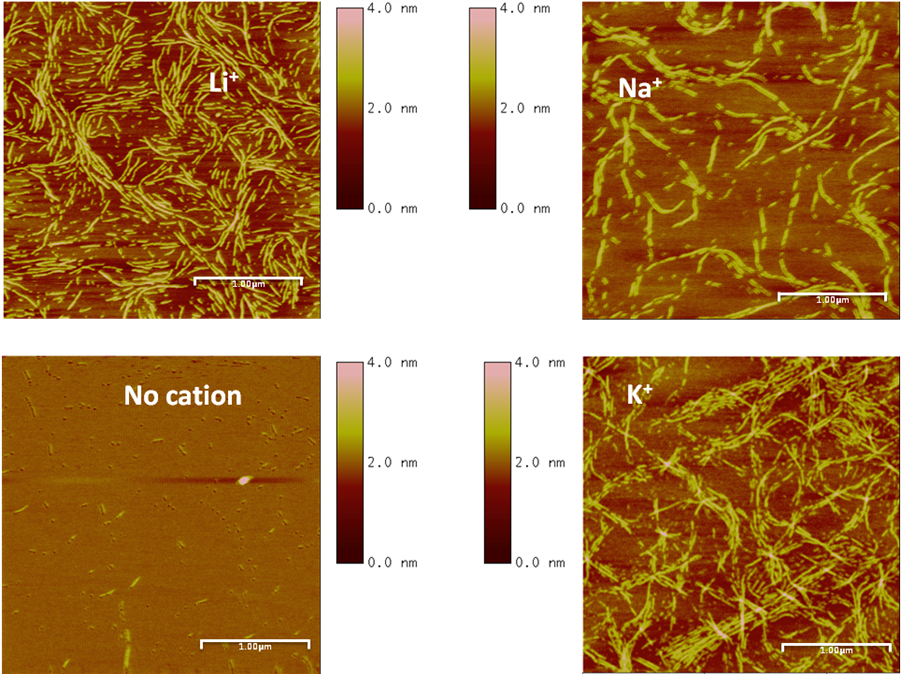


**Figure S1:** Complex formation between c-di-GMP and ProF captured by AFM image. Condition: [c-di-GMP] = 100 μM, [ProF] = 100 μM, [Li^+^, Na^+^, K^+^] = 250 mM in 50 mM Tris-HCl (pH = 7.5), room temperature, incubated for ~12 hr. The mixture was then diluted 20 times to give [c-di-GMP or ProF monomer equivalent] = 5 μM before application to mica plate.


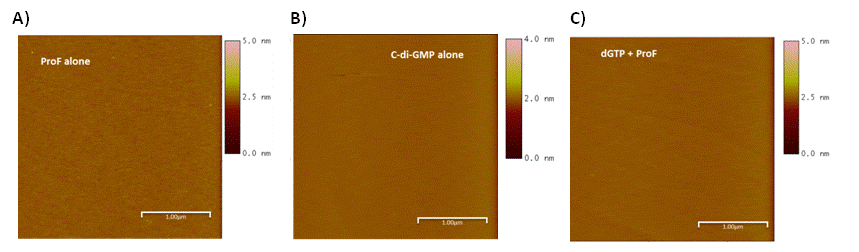


**Figure S2**: C-di-GMP, ProF and dGTP/ProF complex captured by AFM image. Condition: A) [ProF] = 100 μM and [K^+^] = 250 mM in 50 mM Tris-HCl (pH = 7.5) was left to stand overnight (12 hr) and then diluted 20 times to give final concentrations of ProF (5 μM) and K^+^ (12.5 mM), which was deposited on the mica plate for AFM imaging. B) [c-di-GMP] = 100 μM and [K^+^] = 250 mM in 50 mM Tris-HCl (pH = 7.5) was left to stand overnight (12 hr) and then diluted 20 times to give final concentrations of c-di-GMP (5 μM) and K^+^ (12.5 mM), which was deposited on the mica plate for AFM imaging. C) [dGTP] = 100 μM, [ProF] = 100 μM, [K^+^] = 250 mM in 50 mM Tris-HCl (pH = 7.5) was left to stand overnight (12 hr) and then diluted 20 times to give final concentrations of dGTP (5 μM), ProF (5 μM) and K^+^ (12.5 mM), which was deposited on the mica plate for AFM imaging.


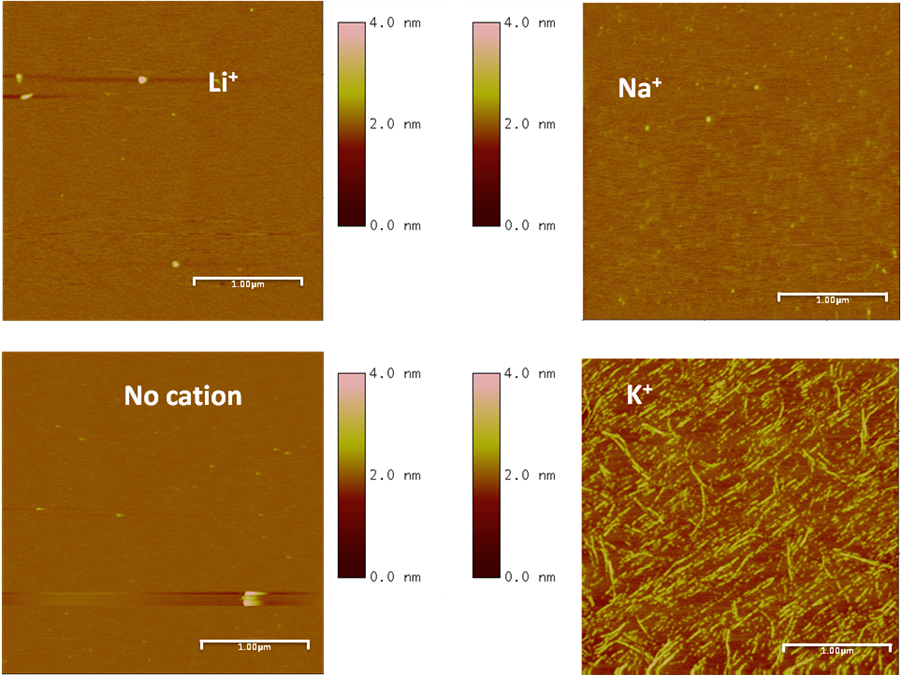


**Figure S3:** Complex formation between c-di-GMP and TO captured by AFM image. Condition: [c-di-GMP] = 100 μM, [TO] = 100 μM, [Li^+^, Na^+^, K^+^] = 250 mM in 50 mM Tris-HCl (pH = 7.5), room temperature, incubated for ~12 hr. The mixture was then diluted 20 times to give [c-di-GMP or ProF monomer equivalent] = 5 μM before application to mica plate.


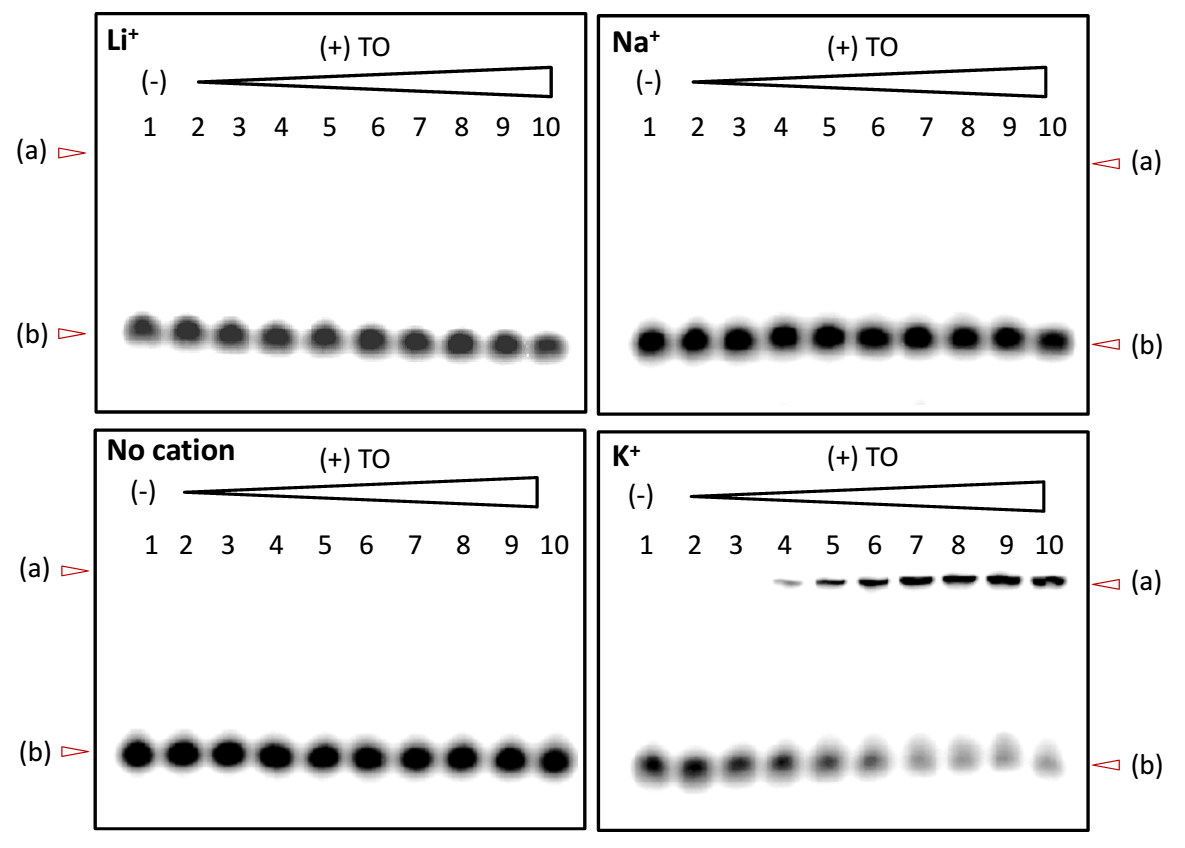


**Figure S4:** Electrophoretic mobility shift assays (EMSA) using native gel. 100 μM unlabeled c-di-GMP and 0.8 nM ^32^P c-di-GMP were incubated with various concentrations of TO in the presence of 250 mM Li^+^, Na^+^, K^+^ or no cation. Final concentration of TO: Lane 1 = 0 μM; Lane 2 = 1 μM; Lane 3 = 5 μM; Lane 4 = 10 μM; Lane 5 = 15 μM; Lane 6 = 20 μM; Lane 7 = 30 μM; Lane 8 = 40 μM; Lane 9 = 50 μM and Lane 10 = 100 μM. (a) = high molecular weight aggregate and (b) = monomeric c-di-GMP.


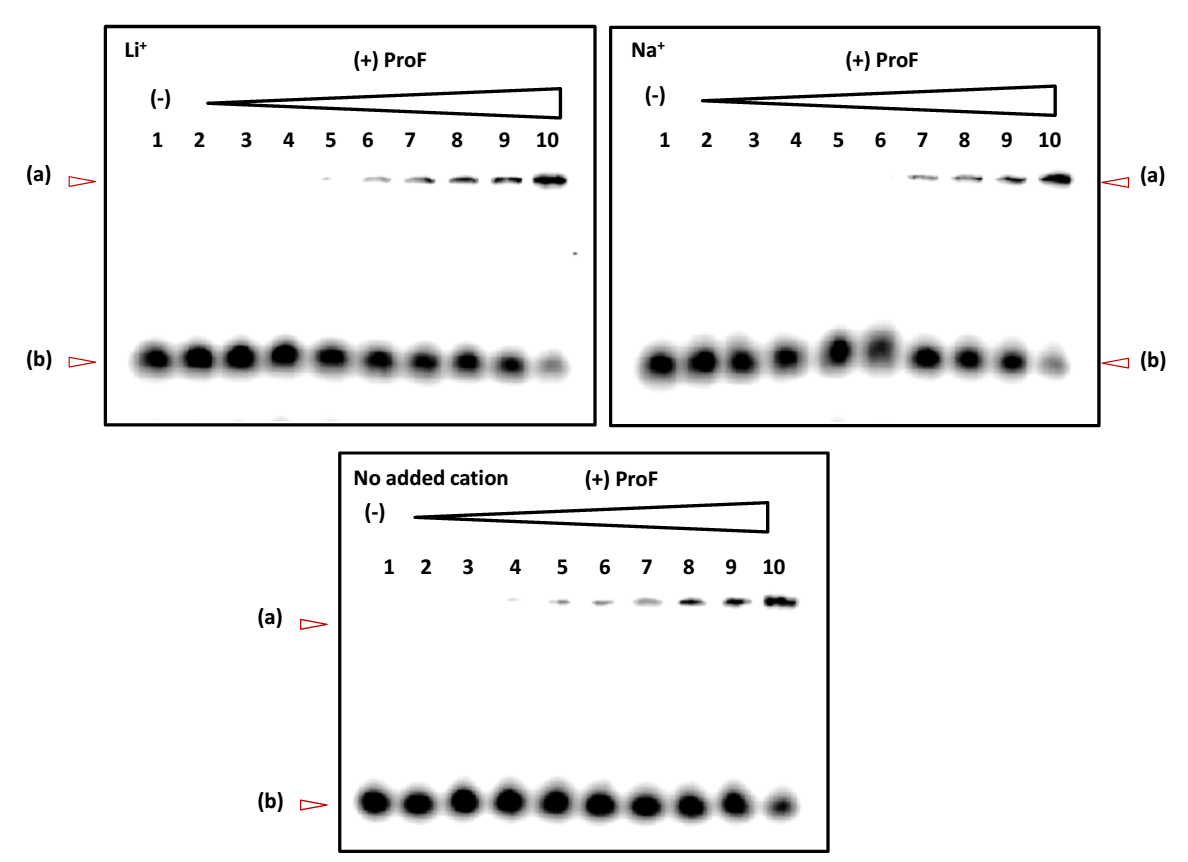


**Figure S5**: EMSA using native gel. 100 μM unlabeled c-di-GMP and 0.8 nM ^32^P c-di-GMP were incubated with various concentrations of ProF in the presence of 250 mM Li^+^, Na^+^ or no cation. Final concentration of ProF: Lane 1 = 0 μM; Lane 2 = 1 μM; Lane 3 = 5 μM; Lane 4 = 10 μM; Lane 5 = 15 μM; Lane 6 = 20 μM; Lane 7 = 30 μM; Lane 8 = 40 μM; Lane 9 = 50 μM and Lane 10 = 100 μM. (a) = high molecular weight aggregate and (b) = monomeric c-di-GMP.


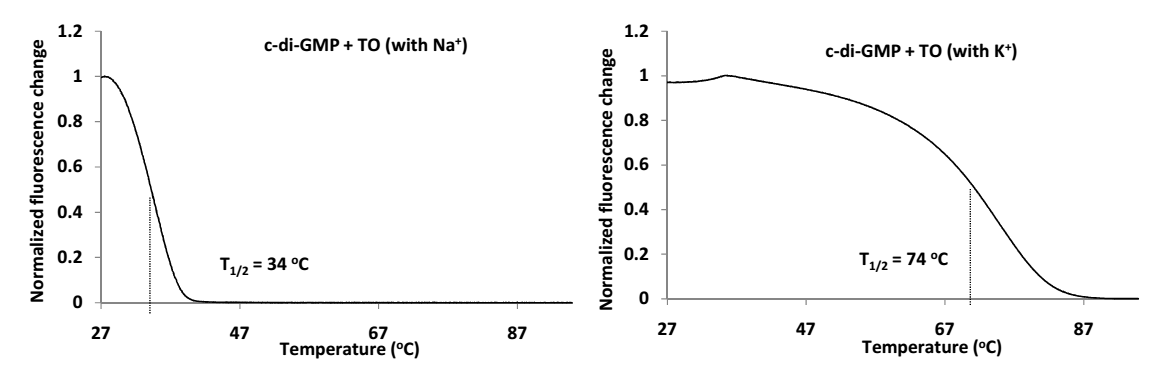


**Figure S6**: Fluorescence melting of the complex formed between c-di-GMP and TO in the presence of NaCl or KCl. Condition: [c-di-GMP] = 100 μM, [ProF or TO] = 10 μM, [Na^+^, K^+^] = 250 mM in 1X MOPS solution (pH = 7.0). Similar T_1/2_ was obtained, compared to the ones in Tris-HCl buffer (see Figure X).


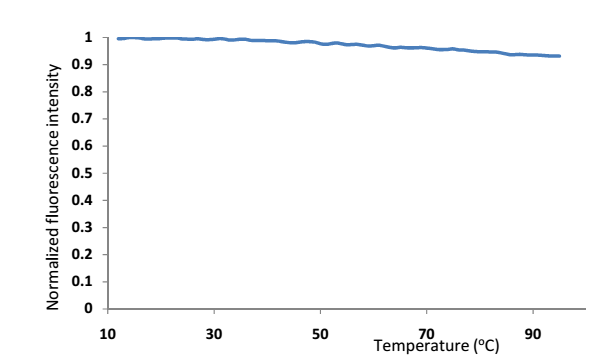


**Figure S7**: Fluorescence melting of ProF alone in the presence of KCl. Condition: [ProF] = 10 μM, [K^+^] = 250 mM in 50 mM Tris-HCl (pH = 7.5).


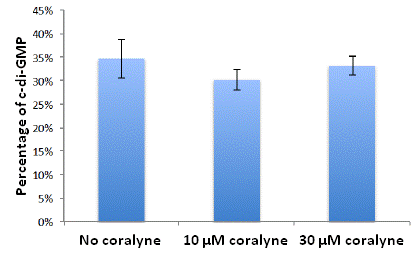


**Figure S8**: YybT cleavage of ^32^P c-di-GMP in the presence and absence of coralyne. Condition: [YybT] = 1.5 μM, [c-di-GMP] = 10 μM (^32^P labeled + unlabeled), [coralyne] = 10 or 30 μM. The cleavage was done at 37 ^o^C for 30 min.
